# Cell-type-specific DNA methylation analysis of the frontal cortices of mutant *Polg1* transgenic mice with neuronal accumulation of deleted mitochondrial DNA

**DOI:** 10.1186/s13041-021-00894-4

**Published:** 2022-01-06

**Authors:** Hiroko Sugawara, Miki Bundo, Takaoki Kasahara, Yutaka Nakachi, Junko Ueda, Mie Kubota-Sakashita, Kazuya Iwamoto, Tadafumi Kato

**Affiliations:** 1 Department of Psychiatry, Kansai Rosai Hospital, Amagasaki, Japan; 2 Department of Psychiatry, Graduate School of Medicine, Osaka University, Osaka, Japan; 3 Department of Molecular Brain Science, Graduate School of Medical Sciences, Kumamoto University, 1-1-1 Honjo, Chuo-ku, Kumamoto-shi, Kumamoto 860-8556 Japan; 4 Career Development Program, RIKEN Center for Brain Science, Saitama, Japan; 5 Department of Psychiatry and Behavior Science, Graduate School of Medicine, Juntendo University, 3-1-3 Hongo, Bunkyo-ku, Tokyo, 113-8431 Japan

**Keywords:** Bipolar disorder, Schizophrenia, DNA methylation, Neuron, Nonneuron, Mitochondrial dysfunction

## Abstract

Bipolar disorder (BD) is a severe psychiatric disorder characterized by repeated conflicting manic and depressive states. In addition to genetic factors, complex gene–environment interactions, which alter the epigenetic status in the brain, contribute to the etiology and pathophysiology of BD. Here, we performed a promoter-wide DNA methylation analysis of neurons and nonneurons derived from the frontal cortices of mutant *Polg1* transgenic (n = 6) and wild-type mice (n = 6). The mutant mice expressed a proofreading-deficient mitochondrial DNA (mtDNA) polymerase under the neuron-specific *CamK2a* promoter and showed BD-like behavioral abnormalities, such as activity changes and altered circadian rhythms. We identified a total of 469 differentially methylated regions (DMRs), consisting of 267 neuronal and 202 nonneuronal DMRs. Gene ontology analysis of DMR-associated genes showed that cell cycle-, cell division-, and inhibition of peptide activity-related genes were enriched in neurons, whereas synapse- and GABA-related genes were enriched in nonneurons. Among the DMR-associated genes, *Trim2* and *Lrpprc* showed an inverse relationship between DNA methylation and gene expression status. In addition, we observed that mutant *Polg1* transgenic mice shared several features of DNA methylation changes in postmortem brains of patients with BD, such as dominant hypomethylation changes in neurons, which include hypomethylation of the molecular motor gene and altered DNA methylation of synapse-related genes in nonneurons. Taken together, the DMRs identified in this study will contribute to understanding the pathophysiology of BD from an epigenetic perspective.

**Supplementary Information:**

The online version contains supplementary material available at 10.1186/s13041-021-00894-4.

## Background

Bipolar disorder (BD) is a severe psychiatric disorder characterized by repeated conflicting states of mania and depression. A recent genome-wide association study revealed that BD is a polygenic disorder caused by multiple genetic risks with small effect sizes [1]. In addition to genetic factors, complex gene–environment interactions may contribute to the high heritability of BD. Epigenetics, such as DNA methylation, is the study of genetic regulation of gene expression without changing the DNA sequences [2]. The majority of DNA methylation occurs at the position of cytosine-guanine (CpG) dinucleotides sequence in mammals. Usually, the extent of methylation at CpG islands, which are located within and around the regulatory promoter regions of genes, correlates with the extent of gene expression. DNA methylation, which is affected by environmental factor, have been considered to reflect gene-environment interactions and contribute to long-lasting alteration of gene expression [3, 4]. A number of DNA methylation studies have suggested to play important roles in the etiology of psychiatric disorders [5, 6].

**Fig. 1:**
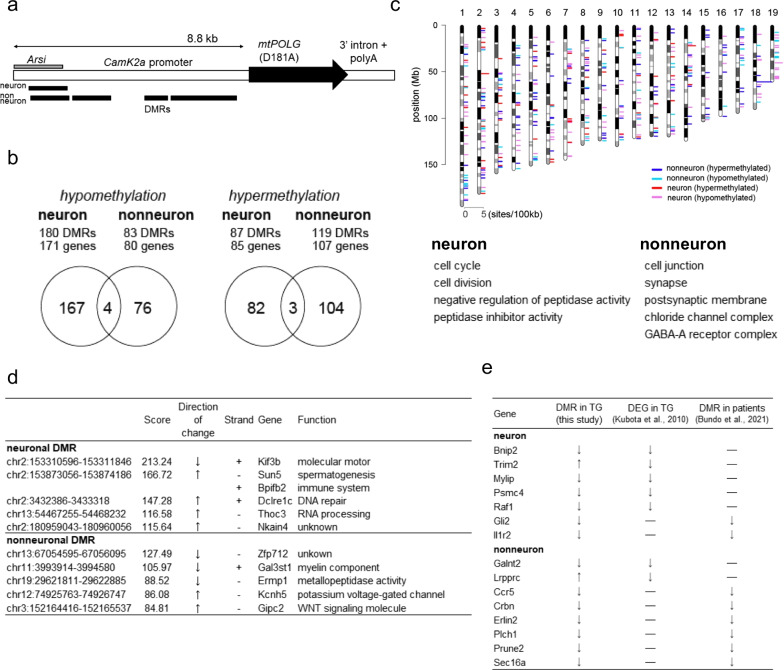
Epigenetic characterization of the brains of mutant *Polg1* Tg mice. **a** The transgene construct of mutant *Polg1* Tg mice. The mutant mice express proofreading-deficit *Polg1* due to a D181A mutation under the neuron-specific *Camk2a* promoter. The upstream region of *Camk2a* (about 8.8 kb) contains a part of the neighboring gene, *Arsi*. Black bars indicate hypermethylated DMRs. Hypermethylated region was detected around *Arsi* in both cell types. Three hypermethylated regions were additionally detected in the *Camk2a* promoter in nonneurons. **b** The number of DMRs and their associated genes in neurons and nonneurons. Venn diagrams are drawn based on the DMR-associated genes. **c** Chromosomal location and top hit significant Gene Ontology terms. See Additional file 3: Table S5 for detailed result of Gene Ontology analysis. **d** Top hit DMRs in neurons and nonneurons. DMRs on the transgene (i.e., *Arsi* and *Camk2a*) or those with no gene symbols were excluded from the list. See Additional file 3: Tables S1–S4.** e** Comparison of DMR-associated genes in the mutant mice with previously reported DEGs in the mutant mice and DMRs in patients with BD. *Arsi* Arylsulfatase I, *BD* bipolar disorder, *Camk2a* calcium/calmodulin-dependent protein kinase type II subunit alpha, *DEG* differentially expressed gene; *DMR* differentially methylated region, *Polg1* DNA polymerase subunit gamma 1

Chronic progressive external ophthalmoplegia, a mitochondrial disease caused by mutations in *POLG1* (DNA polymerase subunit gamma 1) and other genes encoding mitochondrial proteins, often present with comorbid mood disorders [7]. The pathogenic mutations in *POLG1* generally result in the loss of proofreading and/or polymerase activities of mitochondrial DNA (mtDNA) polymerase and cause accumulation of deleted mtDNA in the cells [8, 9]. We have previously generated transgenic (Tg) mice carrying the mutant *Polg1* (D181A) under the neuron-specific *Camk2a* promoter (Fig. 1a) [10]. The mutant mice showed neuron-specific accumulation of deleted mtDNA and behavioral abnormalities potentially capturing some facet of BD, such as recurrent and spontaneous depression-like episodes associated with emotional, vegetative and psychomotor disturbances, and response to antidepressant, mood stabilizer, and electroconvulsive treatments [8, 10, 11]. In this study, we identified differentially methylated regions (DMRs) in the brains of mutant *Polg1* Tg mice and identified candidate DMRs associated with the pathophysiology of BD.

## Methods

We performed promoter-wide DNA methylation analysis of the isolated NeuN + and NeuN- nuclei fraction [12] derived from the frontal cortices of six Tg mice [male (n = 3) and female (n = 3)] and six of their wild-type, sex-matched littermates. We used the same regions with previously performed gene expression analysis of Tg mice [13]. Cell type-specific analysis would be important in the brain tissue [12] and in this Tg mice, as the transgene specifically expresses in neurons. After collecting the densely methylated regions using MBD2B/3L, DNA methylation profiles were obtained with the tiling arrays covering approximately 28,000 mouse promoters (Affymetrix, Santa Clara, CA, USA). DMRs were identified using model-based analysis of tiling array software [14] by comparing Tg mice and wild-type mice datasets. Detailed methods are described in the Additional file 2: Methods section.

## Results and discussion

We identified a total of 469 DMRs, consisting of 267 neuronal and 202 nonneuronal DMRs. Among them, 67.4% (n = 180) and 41.1% (n = 83) were hypomethylated in neurons and nonneurons, respectively (Fig. 1b, Additional file 3: Tables S1–S4). DMRs were distributed throughout the mouse genome (Fig. 1c). Notably, in nonneurons, three DMRs detected in the upstream sequence of *CamK2a* showed hypermethylation in Tg mice compared to nonneurons of wild-type mice (Fig. 1a). In addition, one DMR detected in the *Arsi* showed hypermethylation in both cell types of Tg mice. However, these hypermethylation sites were attributed to changes in the methylation profile of the transgene construct consisting of a long upstream region of the *Camk2a* gene, which contains a part of a neighboring gene, *Arsi*. The fact that the promoter region of *Camk2a* on the transgene was densely methylated in only nonneurons suggested the successful neuron-specific regulation of the mutant *Polg1* expression in Tg mice.

There were a few overlapped DMR-associated genes between neurons and nonneurons, and most of them showed cell-type-specific DNA methylation changes (Fig. 1b, Additional file 1: Discussion). Given that the deleted mtDNA primarily accumulates in neurons [10], the DMRs in nonneurons were considered to be secondarily induced by neuron-nonneuron interactions during development. The top hit list of DMR-associated genes in neurons included hypomethylation of *Kif3b* and hypermethylation of *Sun5*, *Dclre1c*, *Bpifb2*, *Thoc3,* and *Nkain4*. In nonneurons, hypomethylation of *Zfp712*, *Gal3st1*, and *Ermp1*, and hypermethylation of *Kcnh5* and *Gipc2* were included (Fig. 1d).

We performed Gene Ontology analysis using DMR-associated genes. In neurons, we found that cell cycle-, cell division-, and inhibition of peptidase activity-related genes were enriched, whereas cell junction, synapse-related genes, and GABA receptor-related genes were enriched in nonneurons (Fig. 1c and Additional file 3: Table S5).

We then compared the DMR-associated genes and differentially expressed genes (DEGs) that were previously obtained from the frontal cortices of mutant *Polg1* Tg mice [13]. There were seven common genes between neuronal DMR-associated genes and DEGs (*Bnip2, Mylip, Psmc4, Raf1, Trim2, Galnt2*, and *Lrpprc*) (Fig. 1e and Additional file 1: Discussion). The relatively small number of overlapping may be because gene expression profiles were obtained from unsorted bulk cortical tissues. Among the overlapping genes, two genes (*Trim2* in neurons and *Lrpprc* in nonneurons) showed a typical relationship between hypermethylation and downregulation of gene expression (Additional file 1: Discussion).

We then compared DMR-associated genes in mice and postmortem brains of patients with BD [15]. We found hypomethylation of two genes (*Gli2* and *Il1r2*) in neurons and six genes (*Ccr5*, *Crbn*, *Erlin2*, *Plch1*, *Prune2*, and *Sec16A*) in nonneurons (Fig. 1e, Additional file 1: Discussion). We observed that DNA methylation changes in mutant *Polg1* Tg mice shared several features of those identified in the postmortem brains of patients with BD. First, neurons showed more hypomethylation changes than hypermethylation changes in the mutant mice (binominal test, P < 0.0001) and also showed hypomethylation of molecular motor genes in neurons. Second, DMR-associated genes in nonneurons showed clearer enrichment in the synapse and neurotransmitter-related genes than in neurons.

In conclusion, we characterized epigenetic alterations in the neurons and nonneurons of mutant *Polg1* Tg mice. There were some sex-specific behavioral alterations in mutant *Polg1* Tg mice, such as the propensity for spontaneous recurrent hypoactivity in females [11]. Therefore, DMRs may be different between male and female Tg mice. Although further validation and functional assays as well as sex-specific analysis will be needed, the DMRs identified in this study, part of which were shared with BD patients, will contribute to the understanding of the pathophysiology of BD from an epigenetic perspective. Among the DMRs, those associated with gene expression changes, and those common to postmortem brains of BD will be particularly important candidates related to the pathophysiology of BD.

## Supplementary Information

**Additional file 1:** Supplementary Discussion.

**Additional file 2:** Supplementary Methods.

**Additional file 3:** Supplementary Tables.

## Data Availability

Array data has been deposited in the Gene Expression Omnibus as GSE171120. Other datasets used and/or analyzed during the current study are available from the corresponding author on reasonable request.

## Abbreviations

BD: Bipolar disorder
DEG: Differentially expressed gene
DMR: Differentially methylated region
mtDNA: Mitochondrial DNA
Tg mice: Transgenic mice
