# Supplementary material for: Cell-type-specific DNA methylation analysis of the frontal cortices of mutant Polg1 transgenic mice with neuronal accumulation of deleted mitochondrial DNA
Source: Mol Brain. 2022 Jan 6;15:9. doi: 10.1186/s13041-021-00894-4 (PMC8740475; doi:10.1186/s13041-021-00894-4)
Supplement: Supplementary file 1 — Additional file 1: Supplementary Discussion. [file 13041_2021_894_MOESM1_ESM.docx]

**Supplementary Discussion**

**Common DMR-associated genes between neurons and non-neurons**

There were a few common differentially methylated region (DMR) associated genes between neurons and non-neurons in mutant *Polg1* transgenic (Tg) mice (Fig. 1b), consisting of four hypomethylated (*Plch1 Dnase2b*, *Zfp712*, and *Uox*) and three hypermethylated (*Dnaaf6*, *4931428L18Rik,* and *Arsi*) genes.

*Plch1* encodes the phosphoinositide phospholipase C-eta-1. This generates second messengers inositol 1,4,5-trisphosphate and diacylglycerol [1]. Impairment of inositol signaling pathways may underlie the pathophysiology of bipolar disorder (BD) [2], and epigenetic alteration of this gene may be related to the impairment of this pathway. *Dnase2b* (deoxyribonuclease 2 beta) has been reported as one of the candidate genes by a genome-wide association study of alcohol dependence [3], which is known to be highly comorbid with BD [4]. *Zfp712* encodes the zinc finger protein 712. However, its biological function remains unclear. Tg mice overexpressing urate oxidase have been reported to exacerbate the morphological, neurochemical, and functional lesions of the dopaminergic nigrostriatal pathway [5]. Urate possesses both antioxidant and neuroprotective properties and could be related to the pathophysiology of neurodegenerative diseases, such as Parkinson’s disease [6-8].

The commonly hypermethylated gene, *Dnaaf6* (dynein axonemal assembly factor 6) is known to be involved in fertility and development of the reproductive system. However, the functional roles in the brain remain unclear. *4931428L18Rik* encodes a protein with an unknown function. *Arsi* is located in the upstream region of *Camk2a* used in the transgenic construct of mutant *Polg1* Tg mice and was found to be hypermethylated.

**DMR-associated genes and differentially expressed genes (DEGs)**

We compared the DMR-associated genes and differentially expressed genes (DEGs) in mutant *Polg1* Tg mice [9], which were previously obtained from the frontal cortices of Tg mice. There were seven common genes between DMR-associated genes and DEGs (*Bnip2, Mylip, Psmc4, Raf1, Trim2, Galnt2*, and *Lrpprc*) (Fig 1e).

Among the overlapping genes, two genes, tripartite motif containing 2 (*Trim2*) and leucine-rich pentatricopeptide repeat-containing protein (*Lrpprc*), showed a typical inverse relationship between methylation status and gene expression. Deficiencies in *Trim2* levels have been associated with axonal neuropathy, and mutations or loss of *Trim2* leads to progressive neurodegeneration and synaptic loss [10]. *Lrpprc* plays a role in energy metabolism by regulating mitochondrial DNA-coded mRNAs, and mutation of *LRPPRC* causes Leigh syndrome, which is a mitochondrial disease [11]. A recent study reported a significant reduction in *LRPPRC* expression in postmortem brains of patients with Alzheimer’s disease and Parkinson's disease [12].

Among the other five genes, *Raf1* and *Galnt2* may play important roles in the pathophysiology of BD. *Raf1*, which is a Raf kinase, is an upstream activator of the extracellular signal-regulated kinase (ERK) -signaling pathway and contributes to several neuronal functions, including synaptic plasticity, learning, and memory [13-15]. Previous studies have reported that activation of ERKs is significantly reduced in the postmortem brain of depressed suicide victims [16], and electroconvulsive shock activates Raf kinase in the rat brain [17]. These results suggest a possible role for Raf kinases and ERKs in stress and mood modulation [18].

*Galnt2,* encoding GalNAc-T2, has been known as a regulator of high-density lipoprotein cholesterol metabolism. Previous studies have indicated that lipid abnormalities may be associated with the pathophysiology of BD [19, 20], and both DNA methylation and gene expression changes of *GALNT2* have been detected in postmortem brains of patients with BD [21].

**Common DMR-associated genes between human and mouse**

Comparison of DMR-associated genes with those identified in patients with BD [22] revealed two genes (*Gli2* and *Il1r2*) in neurons and six genes (*Ccr5*, *Crbn*, *Erlin2*, *Plch1*, *Prune2*, and *Sec16A*) in non-neurons (Fig 1e). All showed hypomethylation changes in both mice and humans.

Two common genes in neurons were *Gli2* (Gli family zinc finger 2) and *Il1r2* (interleukin-1 receptor type 2). *Gli2* is involved in the development of the dopaminergic system during embryogenesis [23, 24], and the genetic association of *GLI2* with tardive dyskinesia in patients with schizophrenia has been reported [25]. A previous report has suggested that *Il1r2* forms a heteroreceptor complex with the N-methyl-D-aspartate receptor and dopamine D2 receptor and contributes to schizophrenia-like symptoms in mild neuroinflammation [26].

Among the common genes in non-neurons, *Ccr5*, which is a G protein-coupled chemokine receptor that plays an important role in HIV-1 infection, has also been reported to be associated with the inflammatory response in stroke [27]. A recent study reported a possible role of *CCR5* as a biomarker of therapeutic response in patients with major depressive disorder [28]. *Crbn* encodes a celebron protein with ATP-dependent peptidase activity. This is important for the proliferation of NSCs. Mutations in this gene cause recessive nonsyndromic cognitive disability [29]. *Erlin2* encodes a lipid raft-associated protein. Expression of this gene was downregulated in patients with major depressive disorder and in an animal model of depression [30]. *Erlin2* blocks the polyubiquitination and degradation of inositol 1,4,5-trisphosphate receptors [31], suggesting that it could be related to the pathophysiology of mood disorders via alteration of inositol signaling pathways. The importance of *Plch1* in inositol signaling pathways is discussed above. *Prune2* (Prune homolog 2) is a multifunctional protein involved in apoptosis, cell transformation, and synaptic function [32]. *Sec16A* (Sec16 homolog A) encodes a transporter from the endoplasmic reticulum to the Golgi apparatus. The role of these genes in psychiatric disorders remain unknown.

Comparison of DMR-associated genes with those identified in patients with schizophrenia [33] revealed no overlapped genes. We also performed disease association analysis using ToppGene [34], and found no enrichment of DMR-associated genes on the biological pathways related to GWAS of neuropsychiatric disorders including BD.

**References**

1. Hwang JI, Oh YS, Shin KJ, Kim H, Ryu SH, Suh PG. Molecular cloning and characterization of a novel phospholipase C, PLC-eta. Biochem J*.* 2005;389(Pt. 1):181–6

2. Kato T. Current understanding of bipolar disorder: toward integration of biological basis and treatment strategies. Psychiatry Clin Neurosci*.* 2019;73(9):526–40

3. Edenberg HJ, Koller DL, Xuei X, Wetherill L, McClintick JN, Almasy L et al. Genome-wide association study of alcohol dependence implicates a region on chromosome 11. Alcohol Clin Exp Res*.* 2010;34(5):840–52

4. Grunze H, Schaefer M, Scherk H, Born C, Preuss UW. Comorbid bipolar and alcohol use disorder-A therapeutic challenge. Front Psychiatry*.* 2021;12:660432

5. Chen X, Burdett TC, Desjardins CA, Logan R, Cipriani S, Xu Y et al. Disrupted and transgenic urate oxidase alter urate and dopaminergic neurodegeneration. Proc Natl Acad Sci U S A*.* 2013;110(1):300–5

6. Chen X, Wu G, Schwarzschild MA. Urate in Parkinson’s disease: more than a biomarker? Curr Neurol Neurosci Rep*.* 2012;12(4):367–75

7. Constantinescu R, Zetterberg H. Urate as a marker of development and progression in Parkinson’s disease. Drugs Today (Barc)*.* 2011;47(5):369–80

8. Schapira AH, Tolosa E. Molecular and clinical prodrome of Parkinson disease: implications for treatment. Nat Rev Neurol*.* 2010;6(6):309–17

9. Kubota M, Kasahara T, Iwamoto K, Komori A, Ishiwata M, Miyauchi T et al. Therapeutic implications of down-regulation of cyclophilin D in bipolar disorder. Int J Neuropsychopharmacol*.* 2010;13(10):1355–68

10. Ylikallio E, Poyhonen R, Zimon M, De Vriendt E, Hilander T, Paetau A, et al.: Deficiency of the E3 ubiquitin ligase TRIM2 in early-onset axonal neuropathy. Hum Mol Genet 2013; 22(15):2975-2983.

11. Cui J, Wang L, Ren X, Zhang Y, Zhang H: LRPPRC: A Multifunctional Protein Involved in Energy Metabolism and Human Disease. Front Physiol 2019; 10:595.

12. Bennett JP, Jr., Keeney PM: Alzheimer's and Parkinson's brain tissues have reduced expression of genes for mtDNA OXPHOS Proteins, mitobiogenesis regulator PGC-1alpha protein and mtRNA stabilizing protein LRPPRC (LRP130). Mitochondrion 2020; 53:154-157.

13. Grewal SS, York RD, Stork PJ. Extracellular-signal-regulated kinase signalling in neurons. Curr Opin Neurobiol*.* 1999;9(5):544–53

14. Kerkhoff E, Rapp UR. Cell cycle targets of Ras/Raf signalling. Oncogene*.* 1998;17:1457–62

15. Sweatt JD. Mitogen-activated protein kinases in synaptic plasticity and memory. Curr Opin Neurobiol*.* 2004;14(3):311–7

16. Dwivedi Y, Rizavi HS, Roberts RC, Conley RC, Tamminga CA, Pandey GN. Reduced activation and expression of ERK1/2 MAP kinase in the post-mortem brain of depressed suicide subjects. J Neurochem*.* 2001;77(3):916–28

17. Kang UG, Jeon SH, Lee JE, Joo YH, Yi JS, Park JB et al. The activation of B-Raf and Raf-1 after electroconvulsive shock in the rat hippocampus. Neuropharmacology*.* 2000;39(4):703–6

18. Einat H, Yuan P, Gould TD, Li J, Du J, Zhang L et al. The role of the extracellular signal-regulated kinase signaling pathway in mood modulation. J Neurosci*.* 2003;23(19):7311–6

19. Ikeda M, Saito T, Kondo K, Iwata N. Genome-wide association studies of bipolar disorder: A systematic review of recent findings and their clinical implications. Psychiatry Clin Neurosci*.* 2018;72(2):52–63

20. Ikeda M, Takahashi A, Kamatani Y, Okahisa Y, Kunugi H, Mori N et al. A genome-wide association study identifies two novel susceptibility loci and trans population polygenicity associated with bipolar disorder. Mol Psychiatry*.* 2018;23(3):639–47

21. Ho AM, Winham SJ, Armasu SM, Blacker CJ, Millischer V, Lavebratt C et al. Genome-wide DNA methylomic differences between dorsolateral prefrontal and temporal pole cortices of bipolar disorder. J Psychiatr Res*.* 2019;117:45–54

22. Bundo M, Ueda J, Nakachi Y, Kasai K, Kato T, Iwamoto K. Decreased DNA methylation at promoters and gene-specific neuronal hypermethylation in the prefrontal cortex of patients with bipolar disorder. Mol Psychiatry*.* 2021 https://doi.org/10.1038/s41380-021-01079-0

23. Abeliovich A, Hammond R. Midbrain dopamine neuron differentiation: factors and fates. Dev Biol*.* 2007;304(2):447–54

24. Sillitoe RV, Vogel MW. Desire, disease, and the origins of the dopaminergic system. Schizophr Bull*.* 2008;34(2):212–9

25. Greenbaum L, Alkelai A, Rigbi A, Kohn Y, Lerer B. Evidence for association of the GLI2 gene with tardive dyskinesia in patients with chronic schizophrenia. Mov Disord*.* 2010;25(16):2809–17

26. Borroto-Escuela DO, Tarakanov AO, Bechter K, Fuxe K. IL1R2, CCR2, and CXCR4 may form heteroreceptor complexes with NMDAR and D2R: relevance for schizophrenia. Front Psychiatry*.* 2017;8:24

27. Joy MT, Ben Assayag E, Shabashov-Stone D, Liraz-Zaltsman S, Mazzitelli J, Arenas M et al. CCR5 is a therapeutic target for recovery after stroke and traumatic brain injury. Cell*.* 2019;176(5):1143–57.e13

28. Bauer O, Milenkovic VM, Hilbert S, Sarubin N, Weigl J, Bahr LM et al. Association of Chemokine (C-C motif) Receptor 5 and ligand 5 with recovery from major depressive disorder and related neurocognitive impairment. Neuroimmunomodulation*.* 2020. Association of Chemokine;27:152–62

29. Sheereen A, Alaamery M, Bawazeer S, Al Yafee Y, Massadeh S, Eyaid W. A missense mutation in the CRBN gene that segregates with intellectual disability and self-mutilating behaviour in a consanguineous Saudi family. J Med Genet*.* 2017;54(4):236–40

30. Yamagata H, Uchida S, Matsuo K, Harada K, Kobayashi A, Nakashima M et al. Identification of commonly altered genes between in major depressive disorder and a mouse model of depression Sci rep. 2017;7(1):3044

31. Pearce MM, Wormer DB, Wilkens S, Wojcikiewicz RJ. An endoplasmic reticulum (ER) membrane complex composed of SPFH1 and SPFH2 mediates the ER-associated degradation of inositol 1,4,5-trisphosphate receptors. J Biol Chem*.* 2009;284(16):10433–45

32. Cetani F, Pardi E, Marcocci C. Parathyroid carcinoma: a clinical and genetic perspective. Minerva Endocrinol*.* 2018;43(2):144–55

33. Ueda J, Bundo M, Nakachi Y, Kasai K, Kato T, Iwamoto K. Cell type-specific DNA methylation analysis of the prefrontal cortex of patients with schizophrenia. Psychiatry Clin Neurosci. 2021;75:297-299

34. Chen J, Bardes, EE, Aronow BJ, Jegga AG. ToppGene Suite for gene list enrichment analysis and candidate gene prioritization. Nucleic Acids Res. 2009;37:W305-11
