# Supplementary material for: Cell-type-specific DNA methylation analysis of the frontal cortices of mutant Polg1 transgenic mice with neuronal accumulation of deleted mitochondrial DNA
Source: Mol Brain. 2022 Jan 6;15:9. doi: 10.1186/s13041-021-00894-4 (PMC8740475; doi:10.1186/s13041-021-00894-4)
Supplement: Supplementary file 2 — Additional file 2: Supplementary Methods. [file 13041_2021_894_MOESM2_ESM.docx]

**Supplementary Information**

**Materials and methods**

**Animals**

The method for generating mutant *Polg1* transgenic (Tg) mice was performed as previously described [1]. The mutant mice carry *Polg1* lacking proofreading activity due to a D181A mutation. Expression of mutant *Polg1* is regulated under the promoter of calmodulin kinase IIa (*Camk2a*). Male mutant mice were used for mating to avoid possible transmission of mtDNA mutations from the maternal side. The frontal cortices of male (n=3) and female (n=3) Tg mice aged approximately 35 weeks and their wild-type, sex-matched littermates were used in the present study to combine with previously dataset [2]. All experimental procedures involving animals were approved by the RIKEN Center for Brain Science (CBS) Animal Committee.

**Neuronal nuclei isolation**

Neuronal and nonneuronal nuclei fractions were separated by NeuN- based cell sorting [3, 4]. In brief, after homogenization of fresh-frozen brain samples, the nuclear fraction was retrieved by Percoll discontinuous density gradient centrifugation. Anti-NeuN antibody (#MAB377, Millipore, Burlington, MA, USA) conjugated with Alexa Fluor 488 was used for staining. NeuN+ and NeuN- nuclei were sorted using a FACS Aria system (BD Biosciences, Franklin Lakes, NJ, USA).

**Methylation analysis**

Enrichment of methylated DNA was performed using MethylCollector (Active Motif, Carlsbad, CA, USA) according to the manufacturer’s protocol. A total of 100 ng of DNA was used, and methylated DNA was retrieved in 100 µL of elution buffer. Probe preparation and labeling for Affymetrix mouse promoter 1.0R arrays were performed according to the Affymetrix chromatin immunoprecipitation assay protocol (Affymetrix, Santa Clara, CA, USA), as previously described in detail [3]. The array covers 28,000 mouse promoters by 4.6 million 25-mer oligo probes. Each promoter covers approximately 6.0 kb upstream through 2.5 kb downstream of the transcription start site by 35 bp probe spacing.

The total number of array data points were 24, including six Tg and six wild-type animals in two cell types. Array data were deposited in the Gene Expression Omnibus GSE171120. Differentially methylated regions (DMRs) were identified using MAT software [5] by comparing the Tg and control datasets. The parameters used in the MAT were as follows: bandwidth, 300 bp; max gap, 300; min probe, 10; P-value, 1e-5. DMRs on sex chromosomes were excluded from this analysis. DMRs with a MAT score above 1,500 were omitted to avoid possible hybridization artifacts. Annotation was performed using AnnotatR [6]. Gene ontology (GO) analysis was performed using DAVID [7]. The chromosomal location of the DMRs was visualized using CHARANGO software [8].

For comparison with gene expression, we utilized our previously obtained microarray dataset [2]. In brief, gene expression data were obtained from frontal cortices mutant *Polg1* Tg mice aged approximately 35 weeks (n=4) and their wild-type littermates (n=4) with the MG_430 2.0 array (Affymetrix). After normalization by MAS5 (Affymetrix), a two-tailed paired t-test was performed between mutant mice and their littermates. A total of 1,471 probes (p<0.05) were used for comparison in this study. DNA methylation and gene expression profiles were obtained from different animals.

For comparison with DNA methylation data of patients with bipolar disorder, we utilized our previous dataset [9]. In brief, DNA methylation data were obtained in a cell-type-specific manner using a NeuN- based nuclear separation method on the prefrontal cortices of 35 controls and 34 patients with bipolar disorder. DNA methylation data were profiled with a human promoter tiling array and analyzed using the MAT software. DMRs obtained in neurons (n=987) and nonneurons (n=1,296) were used for comparison in this study.

References

1. Kasahara T, Kubota M, Miyauchi T, Noda Y, Mouri A, Nabeshima T, et al.: Mice with neuron-specific accumulation of mitochondrial DNA mutations show mood disorder-like phenotypes. Mol Psychiatry 2006; 11(6):577-593, 523.

2. Kubota M, Kasahara T, Iwamoto K, Komori A, Ishiwata M, Miyauchi T, et al.: Therapeutic implications of down-regulation of cyclophilin D in bipolar disorder. Int J Neuropsychopharmacol 2010; 13(10):1355-1368.

3. Iwamoto K, Bundo M, Ueda J, Oldham MC, Ukai W, Hashimoto E, et al.: Neurons show distinctive DNA methylation profile and higher interindividual variations compared with non-neurons. Genome Res 2011; 21(5):688-696.

4. Bundo M, Kato T, Iwamoto K: Cell Type-Specific DNA Methylation Analysis in Neurons and Glia. In*.*, vol. 105: Springer; 2016: 115-123.

5. Johnson WE, Li W, Meyer CA, Gottardo R, Carroll JS, Brown M, et al.: Model-based analysis of tiling-arrays for ChIP-chip. Proc Natl Acad Sci U S A 2006; 103(33):12457-12462.

6. Cavalcante RG, Sartor MA: annotatr: genomic regions in context. Bioinformatics 2017; 33(15):2381-2383.

7. Huang da W, Sherman BT, Lempicki RA: Systematic and integrative analysis of large gene lists using DAVID bioinformatics resources. Nat Protoc 2009; 4(1):44-57.

8. Nakachi Y, Ishii K, Bundo M, Masuda T, Iwamoto K: Use of the Illumina EPIC methylation array for epigenomic research in the crab-eating macaque (Macaca fascicularis). Neuropsychopharmacol Rep 2020; 40(4):423-426.

9. Bundo M, Ueda J, Nakachi Y, Kasai K, Kato T, Iwamoto K: Decreased DNA methylation at promoters and gene-specific neuronal hypermethylation in the prefrontal cortex of patients with bipolar disorder. Mol Psychiatry 2021.
